# The TSP1-CD47-SIRPα interactome: an immune triangle for the checkpoint era

**DOI:** 10.1007/s00262-023-03465-9

**Published:** 2023-05-22

**Authors:** Enrique Montero, Jeffrey S. Isenberg

**Affiliations:** 1 Department of Diabetes Immunology, City of Hope National Medical Center, 1500 Duarte Road, Duarte, CA 91010 USA; 2 Department of Diabetes Complications and Metabolism, City of Hope National Medical Center, 1500 Duarte Road, Duarte, CA 91010 USA; 3 Arthur Riggs Diabetes and Metabolism Research Institute, City of Hope National Medical Center, 1500 Duarte Road, Duarte, CA 91010 USA

**Keywords:** Thrombospondin-1, CD47, SIRPα, Checkpoint, Cancer, Type 1 diabetes

## Abstract

The use of treatments, such as programmed death protein 1 (PD1) or cytotoxic T lymphocyte-associated antigen 4 (CTLA-4) antibodies, that loosen the natural checks upon immune cell activity to enhance cancer killing have shifted clinical practice and outcomes for the better. Accordingly, the number of antibodies and engineered proteins that interact with the ligand–receptor components of immune checkpoints continue to increase along with their use. It is tempting to view these molecular pathways simply from an immune inhibitory perspective. But this should be resisted. Checkpoint molecules can have other cardinal functions relevant to the development and use of blocking moieties. Cell receptor CD47 is an example of this. CD47 is found on the surface of all human cells. Within the checkpoint paradigm, non-immune cell CD47 signals through immune cell surface signal regulatory protein alpha (SIRPα) to limit the activity of the latter, the so-called *trans* signal. Even so, CD47 interacts with other cell surface and soluble molecules to regulate biogas and redox signaling, mitochondria and metabolism, self-renewal factors and multipotency, and blood flow. Further, the pedigree of checkpoint CD47 is more intricate than supposed. High-affinity interaction with soluble thrombospondin-1 (TSP1) and low-affinity interaction with same-cell SIRPα, the so-called *cis* signal, and non-SIRPα ectodomains on the cell membrane suggests that multiple immune checkpoints converge at and through CD47. Appreciation of this may provide latitude for pathway-specific targeting and intelligent therapeutic effect.

## Introduction

**Fig. 1:**
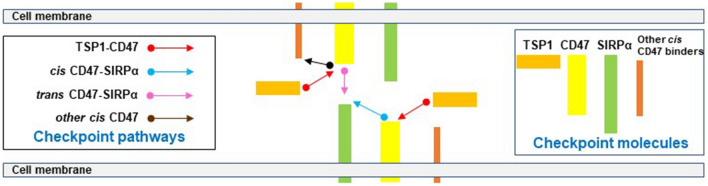
TSP1-CD47-SIRPα immune checkpoints. CD47 is at the center of multiple immune inhibitory checkpoints: (1) TSP1-CD47 checks T, natural killer and dendritic cells, and perhaps macrophages. (2) *Trans* CD47-SIRPα checks macrophage phagocytosis. (3) *Cis* CD47-SIRPα limits inflammation wherever both occur on the same cell. (4) *Cis* CD47-αMβ2 and *cis* CD47-VEGFR-2, labeled other *cis* CD47, check macrophages and T cells, respectively. Acting through cell surface CD47 or SIRPα, the pictured pathways are inhibitory in immune cells. Not pictured are TSP1-SIRPα and ‘reverse’ SIRPα-CD47 signaling [110], which have not been assessed within the checkpoint paradigm. Regarding intracellular effects, SIRPα promotes phosphorylation of SHP1 and SHP2 to quell immune cells [111]. However, SHP1 [112] and SHP2 [113] are activated by other than SIRPα, facts not parsed out in relation to the interactome. Cytoplasmic transmission of the TSP1-CD47 signal is via integrins [114], heterotrimeric G proteins [115], and probably other cell surface molecules. The meager cytoplasmic domain of CD47 encourages this [10]

Checkpoint molecules fine-tune immune cells and prevent inappropriate activity [1, 2]. This informed the development of antibodies that interrupt checkpoint pathways to treat cancer 3. In the U.S.A, over 43% of individuals with cancer are eligible to receive checkpoint blocking molecules [4], which will increase as new agents arrive in the clinic. While stimulating attack of cancer cells, checkpoint blocking antibodies were permissive of immune cell injury toward non-cancer cells [5, 6] and associated with adverse events [7] including insulin-dependent diabetes. The cell surface receptor CD47 is a checkpoint molecule, and companies are developing CD47 blocking antibodies with clinical trials proceeding [8, 9]. CD47 has several natural ligands including secreted thrombospondin-1 (TSP1) [10] and cell membrane signal regulatory protein-alpha (SIRPα) [8]. Through interacting with CD47, both ligands restrain immune cells and foster self-tolerance. TSP1 binds with high affinity to CD47 [11] to suppress T [12], natural killer [13], and dendritic cells [14]. SIRPα binds CD47 with less affinity to restrain phagocytosis [15]. Development of CD47 and SIRPα-binding molecules [16] focused on interrupting the binding between macrophage-displayed SIRPα and non-immune cell-displayed CD47. However, the binding interactions of this trio of molecules are only partly characterized. Current CD47 and SIRPα-blocking agents remain untested in relation to the interaction of TSP1 with CD47, the less studied interaction of TSP1 with SIRPα [17] and in regard to same-cell *cis* CD47-SIRPα. Of relevance, loss of the SIRPα ectodomain, and thus *cis* signaling, altered inflammation in non-immune human cells [18]. Further, human islet endocrine cells, including beta cells, displayed cell surface CD47 but did not display cell surface SIRPα [19], as had been presumed [20]. Put simply, human islet endocrine cells lack *cis* CD47-SIRPα signaling, a finding with possible implications in view of the increased use of CD47 and SIRPα checkpoint blockers. Adding another layer to this narrative, CD47 was linked to metabolism and glucose homeostasis [21]. These findings occasioned the present appraisal of the TSP1-CD47-SIRPα triad (Fig. 1) to understand the possible impact of intersecting these checkpoints.

## Thrombospondin-1

TSP1 is the soluble ligand of the interactome and a trimeric ~ 450 kDa protein secreted by most human cells [22]. It occurs in bodily fluids such as cerebral spinal [23] and plural fluid [24], blood [25], urine [26], and saliva [27] among others. It is also found preformed in platelet alpha granules [28]. Thus, the analysis of soluble TSP1 in body fluids may be complicated by platelet activation. TSP1 modulates cell activity through binding with cell surface receptors including integrins [29], CD36 [30], CD47 [11], and SIRPα [17] and through regulation of growth factors and extracellular matrix [31]. Interestingly, EC retention of TSP1 occurred via the CD47-binding C-terminus of the protein [32], which begs the question if matrix-bound TSP1 can signal through cell surface CD47. Picomolar concentrations of TSP1-activated CD47 [33], suggesting that this interaction dominates under most conditions. In fact, several TSP1-CD36-mediated signals required cross talk with CD47 [34]. The same may be true for certain integrins [35]. Following secretion, TSP1 is scavenged through cell surface internalization [36] and extracellular protease degradation [37]. Additional paracrine effects might be mediated by exosomes which were found decorated with cell surface TSP1, CD47, and SIRPα [38]. In health, TSP1 is found at low non-signaling concentrations (100 ng/ml and less) but is increased with acute and chronic stress, including aging, where it is largely, although not wholly [39], deleterious [31].

## Ligand–receptor interactions

As noted, the secreted protein TSP1 binds with high affinity to CD47 (~ K_D_ of 12 pmol) [11] and to SIRPα [17], although in the latter interaction binding affinities and domain specificity remain to be solved. In fact, exogenous TSP1 blocks CD47 binding to SIRPα. These data were obtained using human protein and cells, an important point as species-specific posttranslational modification of the CD47 ectodomain is essential for TSP1 binding[40]. The human antibody B6H12 blocked TSP1 binding to CD47 and CD47 binding to SIRPα [11]. This is perhaps important, since at least one clinical CD47 checkpoint antibody was inspired by B6H12 [41]. TSP1 is increased by elevated glucose [42], inflammation [43], in experimental [44] and clinical type 1 diabetes [45, 46], and with aging [21]. To wit, aged cells support increased TSP1 binding to CD47 through increased clustering of cell membrane CD47 [47]. This finding speaks to the trimeric structure of TSP1 which theoretically permits a single TSP1 to engage several CD47 simultaneously [48]. Of interest here, fresh human islets secreted substantial amounts of soluble TSP1 [19]. And TSP1 expression was increased by chemotherapy [49] and radiation [50]. Thus, the TSP1-CD47-SIRPα interactome links the endocrine, checkpoint, and cancer worlds.

CD47 is found on all human cells including thymocytes, T and B cells, dendritic cells (DCs), natural killer cells (NKs), monocytes, erythrocytes, and platelets [10]. CD47 is also displayed on cancer cells, and increased expression was associated with worse outcome [51]. Cell membrane SIRPα binds CD47 weakly (K_D_ of ~ 0.5 to 8.0 uM) [52, 53]. Variation in experimental K_D_ notwithstanding, there appears to be no latitude in this for optimum effect. Either too much or too little CD47-SIRPα binding increased immune cell activity and allo-rejection [54]. In canonical *trans* signaling, immune cell displayed SIRPα [52], on binding non-immune cell CD47, suppressed macrophage phagocytosis. The separation between the cell-spanning ectodomains is roughly 14 nm, close to the distance of an immune synapsis [52]. Crystal structure analysis indicated that the distal IgV portions of the ectodomains effected *trans* binding [55]. Consistent with the specificity of the interaction, known SIRPα polymorphisms are outside of the *trans* binding area [55]. And like TSP1 binding CD47, posttranslation ectodomain modifications impacted SIRPα binding to CD47 [56]. However, some human cell types, such as renal tubular epithelial cells [17] and lung alveolar cells [18], among others, simultaneously display CD47 and SIRPα on their cell membrane permitting lateral *cis* interaction and signaling. The SIRPα ectodomain displays two CD47-binding sites [57], one distal and one lateral, although whether this is material to *cis* or *trans* signaling requires additional study. Immunoprecipitation data found SIRPα dimerization in certain immune cells, but this was not likely part of the *trans* CD47–SIRPα interaction [58] consistent with crystal structure evidence of a 1:1 interaction between the ectodomains [55]. Still, the kinetics of the *cis* CD47–SIRPα interaction has not been fully revealed and could vary secondary to competition between the ectodomains.

*Cis* CD47 signaling extends beyond its interaction with same-cell SIRPα. CD47 acted in a *cis* fashion with vascular endothelial growth factor receptor two (VEGFR-2) to maximize the pro-angiogenic signal of VEGF [59]. TSP1 and the C-terminus domain interfered with the CD47-VEGFR-2 *cis* interaction. C*is* CD47-αMβ2-integrin regulated macrophage inflammation [60], although whether TSP1 impacts this is unknown. As well, *cis* CD47-α2β1 integrin signaling regulated T-cell adhesion [61]. More to the point, CD47-blocking agents could alter any *cis* CD47 interaction.

As alluded to, a ligand–receptor interaction between TSP1 and SIRPα was revealed [17]. TSP1, but not the C-terminus domain, bound to SIRPα, co-immunoprecipitated with SIRPα, and at low concentrations, activated SIRPα and its downstream Src homology-2 (SH2) domain containing protein phosphatase SHP1, but not SHP2 [17]. Additionally, a SIRPα-specific blocking antibody abolished TSP1-mediated SIRPα signaling, whereas antisense knockdown or antibody blockade of CD47 did not. However, the implications of this for immune cells await further investigation.

Oxidative stress is a feature of cancer and a consequence of chemotherapy and radiation [62]. CD47 is sensitive to oxidation [63] which may alter *trans* and *cis* ligand–receptor binding. For example, high glucose-mediated oxidative stress enforced CD47-SIRPα binding [64]. It is fair to speculate that diabetes-associated oxidative stress will impact the binding and activity of CD47 and SIRPα checkpoint blockers.

## Immune and inflammatory implications

**Fig. 2:**
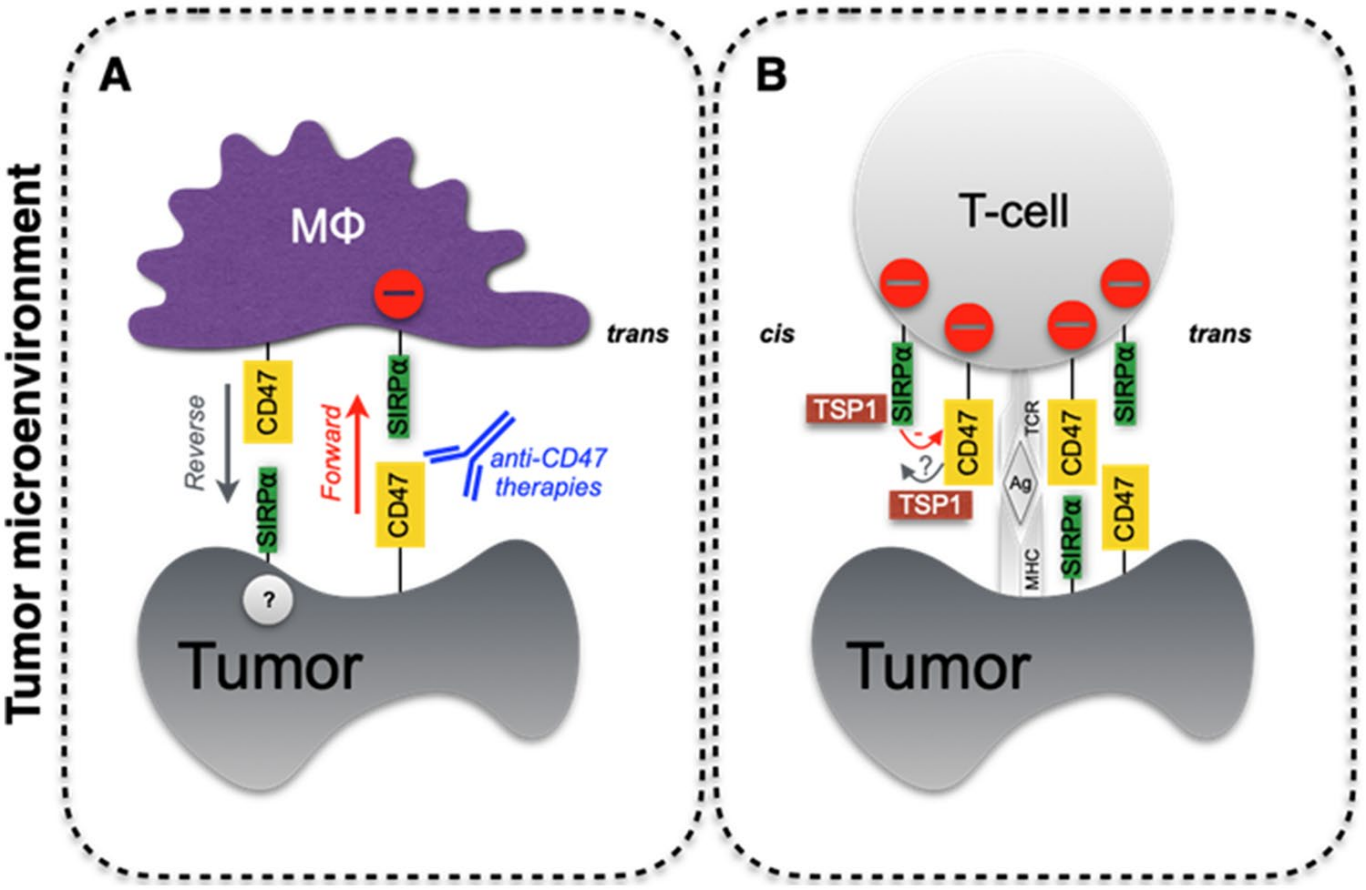
Re-thinking CD47 targeting checkpoint inhibition for cancer immunotherapy. **A** The conventional ‘don’t eat me’ signal mediated by the ‘forward’ negative effect in *trans* of macrophage-displayed CD47. This signal is postulated to be interfered with by clinical blocking anti-CD47 antibodies. The potential ‘reverse’ effect of macrophage CD47 on tumor-expressing SIRPα is unknown. The TSP1-CD47-SIRPα interactome effect on adaptive immunity is ill-defined. **B** Current data support a predominantly negative effect on T cells in *cis*, which may represent a natural mechanism of homeostasis to maintain self-tolerance. It could be reinforced by acting in *trans*, resulting in an additional tumor escape mechanism. And data suggest that TSP1 interferes with CD47 binding to SIRPα presumably in *trans* and *cis*. MΦ, macrophage; TCR, T cell receptor; MHC, major histocompatibility complex; Ag, antigen; mAb, monoclonal antibody

The TSP1-CD47 checkpoint suppresses most immune cells including T cells [65], NKs [13], DCs [14], and macrophages (Fig. 2). The CD47 binding domain of TSP1, but not the SIRPα ectodomain, stimulated human T-cell apoptosis [66]. An oligonucleotide CD47 translation blocker partially decreased total CD47 protein and increased CD8-positive T-cell killing in radiated tumors [67]. Among T cells, the suppressive effect of TSP1 was partly secondary to the inhibition of IL-2 mRNA expression and biogas hydrogen sulfide [68]. This expands upon the known inhibitory effect that TSP1-CD47 has on vascular biogas nitric oxide [33, 69]. And in CD47-null mice, CD4- and CD8-positive T cells were increased [70]. Interestingly, TSP1-treated human T cells showed increased expression of programmed death-ligand 1 (PD-L1) [71] suggesting that TSP1 may co-opt other checkpoints to limit immune activity further.

In human NKs, TSP1 limited transforming growth factor beta (TGF-β)-driven proliferation [72], a process likely involving CD47 [73]. NKs express CD47-SIRPα, which limits cell killing [74]. In line with this, SIRPα-null NKs showed increased cell killing[74]. Interruption of NK cell TSP1-CD47 signaling improved cell homing and increased granzyme B and interferon-γ levels [13]. Although CD47-null NKs had under some circumstances less capacity, consistent with the role CD47 has in metabolic regulation.

TSP1 inhibited human DC activation and cytokine production which was restored by a CD47-blocking antibody [14]. Exhibiting the nuanced effects of TSP1, the CD47-binding C-terminus forced DC tolerance induction, whereas the heparin-binding N-terminus promoted phagocytosis [75]. Here too, a CD47-blocking antibody undid tolerance. TSP1-CD47 signaling decreased human DC differentiation while circulating levels of TSP1 correlated with decreased immune cell response [76]. In this situation, inhibitory SHP1 was activated, suggesting an overlap between TSP1-CD47 and CD47-SIRPα.

Monocytes produced more anti-inflammatory factors in the presence of TSP1, but this involved CD36, an alternative TSP1 receptor [77]. However, the possibility for CD36-CD47 cross talk [78] was not tested. Inflammatory macrophage adhesion was decreased by antibody blockade of TSP1-CD47 [79]. Consistent with these findings, TSP1-null macrophages showed better phagocytosis of sheep red blood cells [80]. Further, *trans* CD47-SIRPα decreased human macrophage phagocytosis [81]. Independent of CD47, the N-terminal domain of TSP1 increased macrophage activation and superoxide production [82]. Whether N- and C-terminus TSP1 signaling simultaneously act upon cells is unknown.

Upending this, tissues [83] and vital organs [84] lacking CD47 fared better than CD47-expressing tissues and organs when transplanted into SIRPα-replete locations. These findings question the primacy of CD47-SIRPα as a checkpoint and delineator of self.

The implications of *cis* CD47-SIRPα signaling for immune cells, especially in correspondence with *trans* signaling, is daunting to sort out (Fig. 2). This is not surprising since there are practical barriers to testing *cis* signaling when the same molecules also interact in a *trans* manner. Macrophage *cis* CD47-SIRPα is a low-affinity interaction (~ K_D_ 1.6 to 2 µM) and acts, separate from *trans* signaling, as a suppressant of phagocytosis [85]. Loss of *cis* signaling by elimination of macrophage cell surface CD47 increased phagocytosis and correlated with less SIRPα phosphorylation. The effect was enhanced when CD47 was blocked on target non-immune cells [85]. Acute CD47 suppression did not alter same-cell SIRPα levels. But this should be determined in somatic human CD47-null T cells, which are employed in the study of these interactions, as compensatory changes might be found. *Cis* CD47-SIRPα signaling was also demonstrated in human epithelial cells [18], albeit from the direction of SIRPα. Loss of functional SIRPα ectodomain in CD47 expressing human lung alveolar epithelial cells increased inflammatory JAK/STAT activity suggesting *cis* CD47-SIRPα limits inflammation in non-immune cells. This was found true for THP-1 human monocytes as well [18]. Thus, loss of same-cell CD47 or SIRPα increases inflammation. SIRPα dimerization was shown in neutrophils [58], but whether this alters *trans* or *cis* interactions with CD47 is unknown. It was proposed that *cis* SIRPα might sequester CD47, rendering non-immune cells ‘CD47 low’ as seen by SIRPα-expressing immune cells [85]. Extending this, *cis* CD47 on immune cells might sequester SIRPα away from the *trans* interaction. One might wonder if *cis*-driven lowering of available CD47 on non-immune cells or available SIRPα on immune cells lowers the overall *trans* signal.

Together these data suggest co-stimulatory roles for TSP1, CD47, and SIRPα. We conceptualize co-stimulatory to include enhancement or suppression of inflammation. Other ligand–receptor pathways such as CD6 showed co-stimulatory effects in T cells based on the binding of ligands CD166 [86] and CD318 [87]. Nonetheless, CD6 was successfully targeted with the immunomodulatory antibody itolizumab [88]. This encourages efforts to optimize therapeutic agents against TSP1, CD47, and SIRPα.

## Interactome pathway disruption

Antibodies to checkpoint CD47 are being tested in numerous trials [89]. The most clinically advanced of these is magrolimab [90]. The CD47 antibody B6H12 was produced by immunizing mice with RGD-binding human placental protein [91] and was a muse for the development of magrolimab [41]. B6H12 blocked RGD binding and immune cell activation [91]. This is important, as TSP1 contains an RGD sequence in its CD47-binding C-terminus [92]. Consequently, magrolimab may block TSP1 binding to CD47. One might surmise that magrolimab also interferes with *cis* CD47-SIRPα (Fig. 2).

Magrolimab binding was tested in rat lymphoblast YB2/0 cells transfected with human CD47. However, rodent cells do not carry out human-specific posttranslational protein modifications that are important for ligand binding [40]. The binding affinity of magrolimab to monomeric human CD47 was estimated to be a K_D_ 8 nM [41], much less than that of TSP1. Magrolimab binding to SIRPα was tested with ELISA and plate-bound protein [41]. Details on several clinical CD47 and SIRPα-blocking molecules that are in or completed trial or that are in development are found in Table 1. Overall, binding information is incomplete. It is fair to say that the therapeutic interruption of checkpoint CD47 remains focused upon *trans* SIRPα. Analysis of *cis* SIRPα, soluble TSP1, and other *cis* CD47-interacting ectodomains is warranted. Similar recommendations apply to SIRPα-targeting molecules.

**Table 1:** CD47- and SIRPα-targeting molecules

Name	Source	Origin	Isotype	Blocks	Kinetics	cis/trans
Magrolimab^1^	Gilead	Humanized	IgG4	CD47-SIRP	4.4E^−11^ M	? / +
AO-176^2^	Arch Oncology	Humanized	IgG2	CD47-SIRP	?	? / +
AO-104	Arch Oncology	Humanized	IgG4	CD47-SIRP	?	? / +
CC90002^3^	Celgene	Humanized	IgG4	CD47-SIRP	?	? / +
SRF231^4^	Surface Oncology	Fully human	IgG4	CD47-SIRP	?	? / ?
IBI188^5^	Innovent	Fully human	IgG4	CD47-SIRP	?	? / +
IBI-322	Innovent	Bi-specific	?	CD47 x PD-L1	?	? / +
TG-1801	TG Therapeutics	Bi-specific	IgG1	CD47 x CD19	?	?
SGN CD47M	Seattle Genetics	Conjugate	?	?	?	?
HX-009	Waterstone Hanxbio	Bi-specific	?	CD47 x PD-1	?	?
IMC-002	Immune-Onco Therapeutics	?	?	?	?	?
AK117^6^	Akeso Biopharma	?	?	CD47-SIRP	1.5E^−10^ M	? / +
STI-6643^7^	Sorrento Therapeutics	Fully human	IgG4	CD47-SIRP	7.6 E^−10^ M	? / +
TKKTLRT-SIRPαFc^8^	Immune-Onco Therapeutics	Collagen-SIRPαFc	?	CD47	1.4E^−9^ M	? / ?

1. Liu J, Wang L, Zhao F, Tseng S, Narayanan C, Shura L, Willingham S, Howard M, Prohaska S, Volkmer J, Chao M, Weissman IL, Majeti R. Pre-clinical development of a humanized anti-CD47 antibody with anti-cancer therapeutic potential. PLoS One. 2015 Sep 21;10(9): e0137345.: 26,390,038

2. Puro RJ, Bouchlaka MN, Hiebsch RR, Capoccia BJ, Donio MJ, Manning PT, Frazier WA, Karr RW, Pereira DS. Development of AO-176, a Next-Generation Humanized Anti-CD47 antibody with novel anticancer properties and negligible red blood cell binding. Mol Cancer Ther. 2020 Mar;19(3):835–846. PMID: 31,879,362

3. Zeidan AM, DeAngelo DJ, Palmer J, Seet CS, Tallman MS, Wei X, Raymon H, Sriraman P, Kopytek S, Bewersdorf JP, Burgess MR, Hege K, Stock W. Phase 1 study of anti-CD47 monoclonal antibody CC-90002 in patients with relapsed/refractory acute myeloid leukemia and high-risk myelodysplastic syndromes. Ann Hematol. 2022 Mar;101(3):557–569. PMID: 34,981,142

4. Peluso MO, Adam A, Armet CM, Zhang L, O'Connor RW, Lee BH, Lake AC, Normant E, Chappel SC, Hill JA, Palombella VJ, Holland PM, Paterson AM. The Fully human anti-CD47 antibody SRF231 exerts dual-mechanism antitumor activity via engagement of the activating receptor CD32a. J Immunother Cancer. 2020 Apr;8(1): e000413. PMID: 32,345,627

5. Ni H, Cao L, Wu Z, Wang L, Zhou S, Guo X, Gao Y, Jing H, Wu M, Liu Y, Ding J, Zhang P, Zhou Y, Chen B, Xiong Y, Sun J, Prinz B, Baruah H, Geoghegan J, Yu M, Wu W, Liu J. Combined strategies for effective cancer immunotherapy with a novel anti-CD47 monoclonal antibody. Cancer Immunol Immunother. 2022 Feb;71(2):353–363. PMID: 34,165,607

6.Qu T, Zhong T, Pang X, Huang Z, Jin C, Wang ZM, Li B, Xia Y. Ligufalimab, a novel anti-CD47 antibody with no hemagglutination demonstrates both monotherapy and combo antitumor activity. J Immunother Cancer. 2022 Nov;10(11): e005517. PMID: 36,450,383

7. Thaker YR, Rivera I, Pedros C, Singh AR, Rivero-Nava L, Zhou H, Swanson BA, Kerwin L, Zhang Y, Gray JD, Kaufmann GF, Ji H, Allen RD, Bresson D. A novel affinity engineered anti-CD47 antibody with improved therapeutic index that preserves erythrocytes and normal immune cells. Front Oncol. 2022 May 19;12: 884,196. PMID: 35,664,753

8. Liu J, Meng Z, Xu T, Kuerban K, Wang S, Zhang X, Fan J, Ju D, Tian W, Huang X, Huang X, Pan D, Chen H, Zhao W, Ye L. A SIRPαFc Fusion Protein Conjugated with the Collagen-Binding Domain for Targeted Immunotherapy of Non-Small Cell Lung Cancer. Front Immunol. 2022 Mar 29;13: 845,217. PMID: 35,422,796

The CD47 ectodomain is also a therapeutic target for peptides [93] and small molecules [94]. Even these agents may overlap *trans* and *cis* CD47-SIRPα, TSP1, and other same-cell CD47 interactions. Screening assays [95] and protocols that encompass the multiple interactions would be useful in the development of CD47- and SIRPα-binding agents. Acute knockdown techniques and existent somatic mutant CD47-null cells [96] may help discriminate between *trans* and *cis* CD47-SIRPα [85].

## Perspectives and interesting questions

Clinical results suggest that this is reasonable to target CD47. But this will likely come with side effects. CD47 on non-immune cells such as red blood cells and platelets soak up CD47 blockers leading to anemia and thrombocytopenia [97]. Because of this, CD47 blockers must be given in large amounts. And CD47 blockers might interfere with blood banking techniques used in cross-matching [98]. Complications from other therapeutic checkpoint molecules are mostly secondary to inflammation and immune injury [99]. Such occurrences may eventually be seen in individuals administered CD47- and SIRPα-targeting agents, especially if used in combination with other checkpoint blockers. Pertinent to this, humanized diabetic mice administered magrolimab lost CD47-overexpressing islet-like grafts and metabolic control [100]. Human islet endocrine cells were found not to have *SIRPA* mRNA or to display cell surface SIRPα protein, even after exposure to diabetes-associated cytokines [19]. It is unknown if the lack of SIRPα is a feature of human endocrine cell types in general and if this deficit sensitizes or protects endocrine cells from CD47 or SIRPα checkpoint blockers. Hypothetically, the lack of SIRPα on islet endocrine cells could increase available CD47 to increase the *trans* CD47-SIRPα signal as further protection from autoimmune injury.

Other means are available to turn down CD47-SIRPα signaling. For example, a translation blocking oligonucleotide to *CD47* mRNA partially lowered total protein, and in combination with radiation, increased T-cell-mediated killing of cancer [67, 101]. While assumed at the time, it is unclear if such an approach decreased cell surface CD47 expression. Alternatively, CRISPR/Cas9 lowered cell surface CD47 and increased phagocytosis [85]. Be that as it may, use of any method targeting TSP1, CD47, and SIRPα should be complemented by the characterization of cell surface molecule copy number (B_max_). And if it is the case that certain knockdown approaches do not substantially alter cell surface CD47 expression, this opens the door for other possible mechanisms of action.

Taking a reverse position, overexpression of cell membrane CD47 [102] was employed to provide a defense against immune cells. Decorating non-animate surfaces with CD47 was also tried [103]. In view of the other homeostatic mechanisms that CD47 impinges upon [10], this strategy may not be benign. For instance, TSP1-CD47 signaling promotes aging in human cells and tissues [104] and animals [31, 105], and limits the Yamanaka self-renewal transcription factors in human cells [106]. In fact, CD47-null cells grown in serum-free medium de-differentiation [107]. Thus, forced CD47 expression may prematurely drive stem cells out of the cell cycle and into senescence [108].

## Conclusion

The TSP1-CD47-SIRPα interactome is a multi-tier check on immune cells. Agents that attempt to intervene on one of these will probably alter or undo other interactome signals. This suggests opportunity to refine the approach to targeting these molecules. Further, checkpoint blocker-sensitive islet endocrine cells are devoid of SIRPα. This occasions the question if this is a protective adaptation. Like beta cells, thyroid cells are derived from endoderm^109^. A closer look at SIRPα in other endocrine organs should prove rewarding.

## References

[1] Keir ME, Butte MJ, Freeman GJ, Sharpe AH (2008). PD-1 and its ligands in tolerance and immunity. Annu Rev Immunol.

[2] Parry RV, Chemnitz JM, Frauwirth KA, Lanfranco AR, Braunstein I, Kobayashi SV, Linsley PS, Thompson CB, Riley JL (2005). CTLA-4 and PD-1 receptors inhibit T-cell activation by distinct mechanisms. Mol Cell Biol.

[3] Hargadon KM, Johnson CE, Williams CJ (2018). Immune checkpoint blockade therapy for cancer: an overview of FDA-approved immune checkpoint inhibitors. Int Immunopharmacol.

[4] Haslam A, Prasad V (2019). Estimation of the percentage of US patients with cancer who are eligible for and respond to checkpoint inhibitor immunotherapy drugs. JAMA Netw Open.

[5] Andres MS, Ramalingam S, Rosen SD, Baksi J, Khattar R, Kirichenko Y, Young K, Yousaf N, Okines A, Huddart R (2022). The spectrum of cardiovascular complications related to immune-checkpoint inhibitor treatment : including myocarditis and the new entity of non inflammatory left ventricular dysfunction. Cardiooncology.

[6] Meraz-Munoz A, Amir E, Ng P, Avila-Casado C, Ragobar C, Chan C, Kim J, Wald R, Kitchlu A (2020) Acute kidney injury associated with immune checkpoint inhibitor therapy: incidence, risk factors and outcomes. J Immunother Cancer 8(1) 10.1136/jitc-2019-000467 PMC7326260 32601079

[7] Fattizzo B, Rampi N, Barcellini W (2022) Hematological and extra-hematological autoimmune complications after checkpoint inhibitors. Pharmaceuticals (Basel) 15(5) 10.3390/ph15050557 PMC9143083 35631383

[8] Matlung HL, Szilagyi K, Barclay NA, van den Berg TK (2017). The CD47-SIRPalpha signaling axis as an innate immune checkpoint in cancer. Immunol Rev.

[9] Kaur S, Cicalese KV, Bannerjee R, Roberts DD (2020). Preclinical and clinical development of therapeutic antibodies targeting functions of CD47 in the tumor microenvironment. Antib Ther.

[10] Kaur S, Isenberg JS, Roberts DD (2021). CD47 (cluster of differentiation 47). Atlas Genet Cytogenet Oncol Haematol.

[11] Isenberg JS, Annis DS, Pendrak ML, Ptaszynska M, Frazier WA, Mosher DF, Roberts DD (2009). Differential interactions of thrombospondin-1, -2, and -4 with CD47 and effects on cGMP signaling and ischemic injury responses. J Biol Chem.

[12] Kaur S, Chang T, Singh SP, Lim L, Mannan P, Garfield SH, Pendrak ML, Soto-Pantoja DR, Rosenberg AZ, Jin S (2014). CD47 signaling regulates the immunosuppressive activity of VEGF in T cells. J Immunol.

[13] Nath PR, Pal-Nath D, Mandal A, Cam MC, Schwartz AL, Roberts DD (2019). Natural killer cell recruitment and activation are regulated by CD47 expression in the tumor microenvironment. Cancer Immunol Res.

[14] Doyen V, Rubio M, Braun D, Nakajima T, Abe J, Saito H, Delespesse G, Sarfati M (2003). Thrombospondin 1 is an autocrine negative regulator of human dendritic cell activation. J Exp Med.

[15] Okazawa H, Motegi S, Ohyama N, Ohnishi H, Tomizawa T, Kaneko Y, Oldenborg PA, Ishikawa O, Matozaki T (2005). Negative regulation of phagocytosis in macrophages by the CD47-SHPS-1 system. J Immunol.

[16] Maute R, Xu J, Weissman IL (2022). CD47-SIRPalpha-targeted therapeutics: status and prospects. Immunooncol Technol.

[17] Yao M, Rogers NM, Csanyi G, Rodriguez AI, Ross MA, St Croix C, Knupp H, Novelli EM, Thomson AW, Pagano PJ (2014). Thrombospondin-1 activation of signal-regulatory protein-alpha stimulates reactive oxygen species production and promotes renal ischemia reperfusion injury. J Am Soc Nephrol.

[18] Londino JD, Gulick D, Isenberg JS, Mallampalli RK (2015). Cleavage of signal regulatory protein alpha (SIRPalpha) enhances inflammatory signaling. J Biol Chem.

[19] Erdem N, Chen KT, Qi M, Zhao Y, Wu X, Garcia I, Ku HT, Montero E, Al-Abdullah IH, Kandeel F et al. (2023) Thrombospondin-1, CD47, and SIRPalpha display cell-specific molecular signatures in human islets and pancreata. Am J Physiol Endocrinol Metab 10.1152/ajpendo.00221.2022 PMC11967708 36791324

[20] Sharp RC, Brown ME, Shapiro MR, Posgai AL, Brusko TM (2021). The immunoregulatory role of the signal regulatory protein family and CD47 signaling pathway in type 1 diabetes. Front Immunol.

[21] Ghimire K, Li Y, Chiba T, Julovi SM, Li J, Ross MA, Straub AC, O'Connell PJ, Ruegg C, Pagano PJ et al. (2020) CD47 Promotes age-associated deterioration in angiogenesis, blood flow and glucose homeostasis. Cells, 9(7) 10.3390/cells9071695 PMC7407670 32679764

[22] Resovi A, Pinessi D, Chiorino G, Taraboletti G (2014). Current understanding of the thrombospondin-1 interactome. Matrix Biol.

[23] Liu BL, Cheng JX, Zhang W, Zhang X, Wang R, Lin H, Huo JL, Cheng H (2010). Quantitative detection of multiple gene promoter hypermethylation in tumor tissue, serum, and cerebrospinal fluid predicts prognosis of malignant gliomas. Neuro Oncol.

[24] Ruiz E, Aleman C, Alegre J, Monasterio J, Segura RM, Armadans L, Vazquez A, Soriano T (2005). Fernandez de Sevilla T: angiogenic factors and angiogenesis inhibitors in exudative pleural effusions. Lung.

[25] Barclay JL, Keshvari S, Whitehead JP, Inder WJ (2016). Development of an enzyme-linked immunosorbent assay for thrombospondin-1 and comparison of human plasma and serum concentrations. Ann Clin Biochem.

[26] Mohammed-Ali Z, Tokar T, Batruch I, Reid S, Tavares-Brum A, Yip P, Cardinal H, Hebert MJ, Li Y, Kim SJ (2019). Urine angiotensin II signature proteins as markers of fibrosis in kidney transplant recipients. Transplantation.

[27] Crombie R, Silverstein RL, MacLow C, Pearce SF, Nachman RL, Laurence J (1998). Identification of a CD36-related thrombospondin 1-binding domain in HIV-1 envelope glycoprotein gp120: relationship to HIV-1-specific inhibitory factors in human saliva. J Exp Med.

[28] Disdier M, Legrand C, Bouillot C, Dubernard V, Pidard D, Nurden AT (1989). Quantitation of platelet fibrinogen and thrombospondin in Glanzmann's thrombasthenia by electroimmunoassay. Thromb Res.

[29] Krutzsch HC, Choe BJ, Sipes JM, Guo N, Roberts DD (1999). Identification of an alpha(3)beta(1) integrin recognition sequence in thrombospondin-1. J Biol Chem.

[30] Magnetto S, Bruno-Bossio G, Voland C, Lecerf J, Lawler J, Delmas P, Silverstein R, Clezardin P (1998). CD36 mediates binding of soluble thrombospondin-1 but not cell adhesion and haptotaxis on immobilized thrombospondin-1. Cell Biochem Funct.

[31] Isenberg JS, Roberts DD (2020). Thrombospondin-1 in maladaptive aging responses: a concept whose time has come. Am J Physiol Cell Physiol.

[32] Adams JC, Bentley AA, Kvansakul M, Hatherley D, Hohenester E (2008). Extracellular matrix retention of thrombospondin 1 is controlled by its conserved C-terminal region. J Cell Sci.

[33] Isenberg JS, Ridnour LA, Dimitry J, Frazier WA, Wink DA, Roberts DD (2006). CD47 is necessary for inhibition of nitric oxide-stimulated vascular cell responses by thrombospondin-1. J Biol Chem.

[34] Isenberg JS, Romeo MJ, Yu C, Yu CK, Nghiem K, Monsale J, Rick ME, Wink DA, Frazier WA, Roberts DD (2008). Thrombospondin-1 stimulates platelet aggregation by blocking the antithrombotic activity of nitric oxide/cGMP signaling. Blood.

[35] Li Z, Calzada MJ, Sipes JM, Cashel JA, Krutzsch HC, Annis DS, Mosher DF, Roberts DD (2002). Interactions of thrombospondins with alpha4beta1 integrin and CD47 differentially modulate T cell behavior. J Cell Biol.

[36] Mikhailenko I, Krylov D, Argraves KM, Roberts DD, Liau G, Strickland DK (1997). Cellular internalization and degradation of thrombospondin-1 is mediated by the amino-terminal heparin binding domain (HBD). High affinity interaction of dimeric HBD with the low density lipoprotein receptor-related protein. J Biol Chem.

[37] Chen CY, Melo E, Jakob P, Friedlein A, Elsasser B, Goettig P, Kueppers V, Delobel F, Stucki C, Dunkley T (2018). N-Terminomics identifies HtrA1 cleavage of thrombospondin-1 with generation of a proangiogenic fragment in the polarized retinal pigment epithelial cell model of age-related macular degeneration. Matrix Biol.

[38] Chauhan S, Danielson S, Clements V, Edwards N, Ostrand-Rosenberg S, Fenselau C (2017). Surface glycoproteins of exosomes shed by myeloid-derived suppressor cells contribute to function. J Proteome Res.

[39] Kaur S, Roberts DD (2023) Why do humans need thrombospondin-1?. J Cell Commun Signal 10.1007/s12079-023-00722-5 PMC10409698 36689135

[40] Kaur S, Kuznetsova SA, Pendrak ML, Sipes JM, Romeo MJ, Li Z, Zhang L, Roberts DD (2011). Heparan sulfate modification of the transmembrane receptor CD47 is necessary for inhibition of T cell receptor signaling by thrombospondin-1. J Biol Chem.

[41] Liu J, Wang L, Zhao F, Tseng S, Narayanan C, Shura L, Willingham S, Howard M, Prohaska S, Volkmer J (2015). Pre-clinical development of a humanized anti-CD47 antibody with anti-cancer therapeutic potential. PLoS ONE.

[42] Wang S, Skorczewski J, Feng X, Mei L, Murphy-Ullrich JE (2004). Glucose up-regulates thrombospondin 1 gene transcription and transforming growth factor-beta activity through antagonism of cGMP-dependent protein kinase repression via upstream stimulatory factor 2. J Biol Chem.

[43] Yesner LM, Huh HY, Pearce SF, Silverstein RL (1996). Regulation of monocyte CD36 and thrombospondin-1 expression by soluble mediators. Arterioscler Thromb Vasc Biol.

[44] Kivela R, Silvennoinen M, Touvra AM, Lehti TM, Kainulainen H, Vihko V (2006). Effects of experimental type 1 diabetes and exercise training on angiogenic gene expression and capillarization in skeletal muscle. FASEB J.

[45] Bayraktar M, Dundar S, Kirazli S, Teletar F (1994). Platelet factor 4, beta-thromboglobulin and thrombospondin levels in type I diabetes mellitus patients. J Int Med Res.

[46] Wahab NA, Schaefer L, Weston BS, Yiannikouris O, Wright A, Babelova A, Schaefer R, Mason RM (2005). Glomerular expression of thrombospondin-1, transforming growth factor beta and connective tissue growth factor at different stages of diabetic nephropathy and their interdependent roles in mesangial response to diabetic stimuli. Diabetologia.

[47] Wang F, Liu YH, Zhang T, Gao J, Xu Y, Xie GY, Zhao WJ, Wang H, Yang YG (2020). Aging-associated changes in CD47 arrangement and interaction with thrombospondin-1 on red blood cells visualized by super-resolution imaging. Aging Cell.

[48] Bazzazi H, Zhang Y, Jafarnejad M, Isenberg JS, Annex BH, Popel AS (2018). Computer simulation of TSP1 inhibition of VEGF-Akt-eNOS: an angiogenesis triple threat. Front Physiol.

[49] Guillon J, Petit C, Moreau M, Toutain B, Henry C, Roche H, Bonichon-Lamichhane N, Salmon JP, Lemonnier J, Campone M (2019). Regulation of senescence escape by TSP1 and CD47 following chemotherapy treatment. Cell Death Dis.

[50] Carpizo DR, Gensure RH, Yu X, Gendel VM, Greene SJ, Moore DF, Jabbour SK, Nosher JL (2014). Pilot study of angiogenic response to yttrium-90 radioembolization with resin microspheres. J Vasc Interv Radiol.

[51] Catalan R, Orozco-Morales M, Hernandez-Pedro NY, Guijosa A, Colin-Gonzalez AL, Avila-Moreno F, Arrieta O (2020). CD47-SIRPalpha axis as a biomarker and therapeutic target in cancer: current perspectives and future challenges in nonsmall cell lung cancer. J Immunol Res.

[52] Hatherley D, Harlos K, Dunlop DC, Stuart DI, Barclay AN (2007). The structure of the macrophage signal regulatory protein alpha (SIRPalpha) inhibitory receptor reveals a binding face reminiscent of that used by T cell receptors. J Biol Chem.

[53] Vernon-Wilson EF, Kee WJ, Willis AC, Barclay AN, Simmons DL, Brown MH (2000). CD47 is a ligand for rat macrophage membrane signal regulatory protein SIRP (OX41) and human SIRPalpha 1. Eur J Immunol.

[54] Dai H, Friday AJ, Abou-Daya KI, Williams AL, Mortin-Toth S, Nicotra ML, Rothstein DM, Shlomchik WD, Matozaki T, Isenberg JS et al. (2017) Donor SIRPalpha polymorphism modulates the innate immune response to allogeneic grafts. Sci Immunol 2(12) 10.1126/sciimmunol.aam6202 PMC5653256 28783664

[55] Hatherley D, Graham SC, Turner J, Harlos K, Stuart DI, Barclay AN (2008). Paired receptor specificity explained by structures of signal regulatory proteins alone and complexed with CD47. Mol Cell.

[56] van den Nieuwenhof IM, de Renardel Lavalette C, Diaz N, van Die I, van den Berg TK (2001). Differential galactosylation of neuronal and haematopoietic signal regulatory protein-alpha determines its cellular binding-specificity. J Cell Sci.

[57] Lee WY, Weber DA, Laur O, Severson EA, McCall I, Jen RP, Chin AC, Wu T, Gernert KM, Parkos CA (2007). Novel structural determinants on SIRP alpha that mediate binding to CD47. J Immunol.

[58] Lee WY, Weber DA, Laur O, Stowell SR, McCall I, Andargachew R, Cummings RD, Parkos CA (2010). The role of cis dimerization of signal regulatory protein alpha (SIRPalpha) in binding to CD47. J Biol Chem.

[59] Kaur S, Martin-Manso G, Pendrak ML, Garfield SH, Isenberg JS, Roberts DD (2010). Thrombospondin-1 inhibits VEGF receptor-2 signaling by disrupting its association with CD47. J Biol Chem.

[60] Podolnikova NP, Key S, Wang X, Ugarova TP (2023). The CIS association of CD47 with integrin Mac-1 regulates macrophage responses by stabilizing the extended integrin conformation. J Biol Chem.

[61] Barazi HO, Li Z, Cashel JA, Krutzsch HC, Annis DS, Mosher DF, Roberts DD (2002). Regulation of integrin function by CD47 ligands. Differential effects on alpha vbeta 3 and alpha 4beta1 integrin-mediated adhesion. J Biol Chem.

[62] Benz CC, Yau C (2008). Ageing, oxidative stress and cancer: paradigms in parallax. Nat Rev Cancer.

[63] Burger P, Hilarius-Stokman P, de Korte D, van den Berg TK, van Bruggen R (2012). CD47 functions as a molecular switch for erythrocyte phagocytosis. Blood.

[64] Maile LA, Capps BE, Miller EC, Allen LB, Veluvolu U, Aday AW, Clemmons DR (2008). Glucose regulation of integrin-associated protein cleavage controls the response of vascular smooth muscle cells to insulin-like growth factor-I. Mol Endocrinol.

[65] Rodriguez-Jimenez P, Chicharro P, Llamas-Velasco M, Cibrian D, Trigo-Torres L, Vara A, Jimenez-Fernandez M, Sevilla-Montero J, Calzada MJ, Sanchez-Madrid F (2019). Thrombospondin-1/CD47 Interaction regulates Th17 and Treg differentiation in psoriasis. Front Immunol.

[66] Lamy L, Ticchioni M, Rouquette-Jazdanian AK, Samson M, Deckert M, Greenberg AH, Bernard A (2003). CD47 and the 19 kDa interacting protein-3 (BNIP3) in T cell apoptosis. J Biol Chem.

[67] Soto-Pantoja DR, Terabe M, Ghosh A, Ridnour LA, DeGraff WG, Wink DA, Berzofsky JA, Roberts DD (2014). CD47 in the tumor microenvironment limits cooperation between antitumor T-cell immunity and radiotherapy. Cancer Res.

[68] Miller TW, Kaur S, Ivins-O'Keefe K, Roberts DD (2013). Thrombospondin-1 is a CD47-dependent endogenous inhibitor of hydrogen sulfide signaling in T cell activation. Matrix Biol.

[69] Isenberg JS, Ridnour LA, Perruccio EM, Espey MG, Wink DA, Roberts DD (2005). Thrombospondin-1 inhibits endothelial cell responses to nitric oxide in a cGMP-dependent manner. Proc Natl Acad Sci U S A.

[70] Engelbertsen D, Autio A, Verwilligen RAF, Depuydt MAC, Newton G, Rattik S, Levinsohn E, Saggu G, Jarolim P, Wang H (2019). Increased lymphocyte activation and atherosclerosis in CD47-deficient mice. Sci Rep.

[71] Liu Z, Wen J, Hu F, Wang J, Hu C, Zhang W (2022). Thrombospondin-1 induced programmed death-ligand 1-mediated immunosuppression by activating the STAT3 pathway in osteosarcoma. Cancer Sci.

[72] Pierson BA, Gupta K, Hu WS, Miller JS (1996). Human natural killer cell expansion is regulated by thrombospondin-mediated activation of transforming growth factor-beta 1 and independent accessory cell-derived contact and soluble factors. Blood.

[73] Soto-Pantoja DR, Shih HB, Maxhimer JB, Cook KL, Ghosh A, Isenberg JS, Roberts DD (2014). Thrombospondin-1 and CD47 signaling regulate healing of thermal injury in mice. Matrix Biol.

[74] Deuse T, Hu X, Agbor-Enoh S, Jang MK, Alawi M, Saygi C, Gravina A, Tediashvili G, Nguyen VQ, Liu Y et al. (2021) The SIRPalpha-CD47 immune checkpoint in NK cells. J Exp Med, 218(3) 10.1084/jem.20200839 PMC7802363 33416832

[75] Tabib A, Krispin A, Trahtemberg U, Verbovetski I, Lebendiker M, Danieli T, Mevorach D (2009). Thrombospondin-1-N-terminal domain induces a phagocytic state and thrombospondin-1-C-terminal domain induces a tolerizing phenotype in dendritic cells. PLoS ONE.

[76] Bandyopadhyay G, Bandyopadhyay S, Bankey PE, Miller-Graziano CL (2014). Elevated postinjury thrombospondin 1-CD47 triggering aids differentiation of patients' defective inflammatory CD1a+dendritic cells. J Leukoc Biol.

[77] Fang LL, Yu HQ, Wu RJ, He C, Li M, Yan H, Li JJ, Wang S, Liu ZG, Liu ZJ (2015). Thrombospondin 1 modulates monocyte properties to suppress intestinal mucosal inflammation. J Innate Immun.

[78] Miller TW, Isenberg JS, Shih HB, Wang Y, Roberts DD (2010). Amyloid-beta inhibits No-cGMP signaling in a CD36- and CD47-dependent manner. PLoS ONE.

[79] Yamauchi Y, Kuroki M, Imakiire T, Uno K, Abe H, Beppu R, Yamashita Y, Kuroki M, Shirakusa T (2002). Opposite effects of thrombospondin-1 via CD36 and CD47 on homotypic aggregation of monocytic cells. Matrix Biol.

[80] McMaken S, Exline MC, Mehta P, Piper M, Wang Y, Fischer SN, Newland CA, Schrader CA, Balser SR, Sarkar A (2011). Thrombospondin-1 contributes to mortality in murine sepsis through effects on innate immunity. PLoS ONE.

[81] Jaiswal S, Chao MP, Majeti R, Weissman IL (2010). Macrophages as mediators of tumor immunosurveillance. Trends Immunol.

[82] Martin-Manso G, Galli S, Ridnour LA, Tsokos M, Wink DA, Roberts DD (2008). Thrombospondin 1 promotes tumor macrophage recruitment and enhances tumor cell cytotoxicity of differentiated U937 cells. Cancer Res.

[83] Isenberg JS, Pappan LK, Romeo MJ, Abu-Asab M, Tsokos M, Wink DA, Frazier WA, Roberts DD (2008). Blockade of thrombospondin-1-CD47 interactions prevents necrosis of full thickness skin grafts. Ann Surg.

[84] Chen M, Wang Y, Wang H, Sun L, Fu Y, Yang YG (2019). Elimination of donor CD47 protects against vascularized allograft rejection in mice. Xenotransplantation.

[85] Hayes BH, Tsai RK, Dooling LJ, Kadu S, Lee JY, Pantano D, Rodriguez PL, Subramanian S, Shin JW, Discher DE (2020) Macrophages show higher levels of engulfment after disruption of cis interactions between CD47 and the checkpoint receptor SIRPalpha. J Cell Sci 133(5) 10.1242/jcs.237800 PMC7064788 31964705

[86] Nair P, Melarkode R, Rajkumar D, Montero E (2010). CD6 synergistic co-stimulation promoting proinflammatory response is modulated without interfering with the activated leucocyte cell adhesion molecule interaction. Clin Exp Immunol.

[87] Enyindah-Asonye G, Li Y, Ruth JH, Spassov DS, Hebron KE, Zijlstra A, Moasser MM, Wang B, Singer NG, Cui H (2017). CD318 is a ligand for CD6. Proc Natl Acad Sci U S A.

[88] Krupashankar DS, Dogra S, Kura M, Saraswat A, Budamakuntla L, Sumathy TK, Shah R, Gopal MG, Narayana Rao T, Srinivas CR (2014). Efficacy and safety of itolizumab, a novel anti-CD6 monoclonal antibody, in patients with moderate to severe chronic plaque psoriasis: results of a double-blind, randomized, placebo-controlled, phase-III study. J Am Acad Dermatol.

[89] Veillette A, Tang Z (2019). Signaling regulatory protein (SIRP)alpha-CD47 blockade joins the ranks of immune checkpoint inhibition. J Clin Oncol.

[90] Swoboda DM, Sallman DA (2020). The promise of macrophage directed checkpoint inhibitors in myeloid malignancies. Best Pract Res Clin Haematol.

[91] Gresham HD, Goodwin JL, Allen PM, Anderson DC, Brown EJ (1989). A novel member of the integrin receptor family mediates Arg-Gly-Asp-stimulated neutrophil phagocytosis. J Cell Biol.

[92] Gao AG, Lindberg FP, Finn MB, Blystone SD, Brown EJ, Frazier WA (1996). Integrin-associated protein is a receptor for the C-terminal domain of thrombospondin. J Biol Chem.

[93] Jalil AR, Tobin MP, Discher DE (2022). Suppressing or enhancing macrophage engulfment through the use of CD47 and related peptides. Bioconjug Chem.

[94] Miller TW, Amason JD, Garcin ED, Lamy L, Dranchak PK, Macarthur R, Braisted J, Rubin JS, Burgess TL, Farrell CL (2019). Quantitative high-throughput screening assays for the discovery and development of SIRPalpha-CD47 interaction inhibitors. PLoS ONE.

[95] Burgess TL, Amason JD, Rubin JS, Duveau DY, Lamy L, Roberts DD, Farrell CL, Inglese J, Thomas CJ, Miller TW (2020). A homogeneous SIRPalpha-CD47 cell-based, ligand-binding assay: utility for small molecule drug development in immuno-oncology. PLoS ONE.

[96] Ticchioni M, Raimondi V, Lamy L, Wijdenes J, Lindberg FP, Brown EJ, Bernard A (2001). Integrin-associated protein (CD47/IAP) contributes to T cell arrest on inflammatory vascular endothelium under flow. FASEB J.

[97] Sikic BI, Lakhani N, Patnaik A, Shah SA, Chandana SR, Rasco D, Colevas AD, O'Rourke T, Narayanan S, Papadopoulos K (2019). First-in-human, first-in-class phase i trial of the anti-CD47 antibody Hu5F9-G4 in patients with advanced cancers. J Clin Oncol.

[98] Brierley CK, Staves J, Roberts C, Johnson H, Vyas P, Goodnough LT, Murphy MF (2019). The effects of monoclonal anti-CD47 on RBCs, compatibility testing, and transfusion requirements in refractory acute myeloid leukemia. Transfusion.

[99] Konig D, Laubli H (2021). Mechanisms of immune-related complications in cancer patients treated with immune checkpoint inhibitors. Pharmacology.

[100] Hu X, Gattis C, Olroyd AG, Friera AM, White K, Young C, Basco R, Lamba M, Wells F, Ankala R (2023). Human hypoimmune primary pancreatic islets avoid rejection and autoimmunity and alleviate diabetes in allogeneic humanized mice. Sci Transl Med.

[101] Maxhimer JB, Soto-Pantoja DR, Ridnour LA, Shih HB, Degraff WG, Tsokos M, Wink DA, Isenberg JS, Roberts DD (2009) Radioprotection in normal tissue and delayed tumor growth by blockade of CD47 signaling. Sci Transl Med, 1(3):3ra7. 10.1126/scitranslmed.3000139 PMC2811586 20161613

[102] Shrestha P, Batra L, Tariq Malik M, Tan M, Yolcu ES, Shirwan H (2020). Immune checkpoint CD47 molecule engineered islets mitigate instant blood-mediated inflammatory reaction and show improved engraftment following intraportal transplantation. Am J Transplant.

[103] Choi MJ, Choi KC, Lee DH, Jeong HY, Kang SJ, Kim MW, Jeong IH, You YM, Lee JS, Lee YK (2022). EGF receptor-targeting cancer therapy using CD47-engineered cell-derived nanoplatforms. Nanotechnol Sci Appl.

[104] Frazier EP, Isenberg JS, Shiva S, Zhao L, Schlesinger P, Dimitry J, Abu-Asab MS, Tsokos M, Roberts DD, Frazier WA (2011). Age-dependent regulation of skeletal muscle mitochondria by the thrombospondin-1 receptor CD47. Matrix Biol.

[105] Isenberg JS, Hyodo F, Pappan LK, Abu-Asab M, Tsokos M, Krishna MC, Frazier WA, Roberts DD (2007). Blocking thrombospondin-1/CD47 signaling alleviates deleterious effects of aging on tissue responses to ischemia. Arterioscler Thromb Vasc Biol.

[106] Rogers NM, Zhang ZJ, Wang JJ, Thomson AW, Isenberg JS (2016). CD47 regulates renal tubular epithelial cell self-renewal and proliferation following renal ischemia reperfusion. Kidney Int.

[107] Kaur S, Soto-Pantoja DR, Stein EV, Liu C, Elkahloun AG, Pendrak ML, Nicolae A, Singh SP, Nie Z, Levens D (2013). Thrombospondin-1 signaling through CD47 inhibits self-renewal by regulating c-Myc and other stem cell transcription factors. Sci Rep.

[108] Meijles DN, Sahoo S, Al Ghouleh I, Amaral JH, Bienes-Martinez R, Knupp HE, Attaran S, Sembrat JC, Nouraie SM, Rojas MM et al. (2017) The matricellular protein TSP1 promotes human and mouse endothelial cell senescence through CD47 and Nox1. Sci Signal, 10(501) 10.1126/scisignal.aaj1784 PMC5679204 29042481

[109] Zorn AM, Wells JM (2009). Vertebrate endoderm development and organ formation. Annu Rev Cell Dev Biol.

[110] Sarfati M, Fortin G, Raymond M, Susin S (2008). CD47 in the immune response: role of thrombospondin and SIRP-alpha reverse signaling. Curr Drug Targets.

[111] Cant CA, Ullrich A (2001). Signal regulation by family conspiracy. Cell Mol Life Sci.

[112] Yousefi S, Simon HU (2003). SHP-1: a regulator of neutrophil apoptosis. Semin Immunol.

[113] Qu CK (2000). The SHP-2 tyrosine phosphatase: signaling mechanisms and biological functions. Cell Res.

[114] Brown EJ, Frazier WA (2001). Integrin-associated protein (CD47) and its ligands. Trends Cell Biol.

[115] Frazier WA, Gao AG, Dimitry J, Chung J, Brown EJ, Lindberg FP, Linder ME (1999). The thrombospondin receptor integrin-associated protein (CD47) functionally couples to heterotrimeric Gi. J Biol Chem.

